# Account of Diseases in an Eastern District of London

**Published:** 1802-12-01

**Authors:** 


*Account of Diseases in an Eaftern Diftrict of London,*

From October 20 to November 20, 1802.

| | |
|---|---|
| ACUTE DISEASES. | |
| Typhus Mitior - - - - | 4 |
| Peripneumonia - - - - | 3 |
| Dyfenteria - - - - - | 4 |
| Variola - - - - - - - | 3 |
| Rheumatifmus Acutus - | 2 |
| Scarlatina Anginofa - - | 3 |
| CHRONIC DISEASES. | |
| Dyfpnœa - - - - - - | 12 |
| Tuffis - - - - - - - | 14 |
| Phthifis Pulmonalis - - | 3 |
| Pleurodyne - - - - | 2 |
| Hydrothorax - - - - | 5 |
| Afcites - - - - - - . | 2 |
| Anafarca - - - - - | 4 |
| Cephalalgia - - - - | 6 |
| Vertigo - - - - - - - | 2 |
| Ophthalmia - - - - | 3 |
| Paralyfis - - - - - - | 1 |
| Chlorofis - - - - - - | 7 |
| Amenorrhœa - - - - | 9 |
| Gaftrodynia - - - - - | 4 |
| Diarrhœa - - - - - | 3 |
| Hæmorrhois - - - - - | 2 |
| Fluor Albus - - - - - | 6 |
| Dyfuria - - - - - - - | 2 |
| PUERPERAL DISEASES. | |
| Menorrahagia Lochialis - | 4 |
| Maftodynia - - - - - - | 2 |
| Dolores poft Partum - - | 5 |
| INFANTILE DISEASES. | |
| Ophthalmia - - - - | 3 |
| Aphthæ - - - - - - - | 5 |
| Tinea Capitis - - - - - | 4 |

Numerous inftances of Scarlatina Anginofa have occurred. This difeafe is not uncommon at this feafon of the year, and is frequently attended with more alarming fymptoms than in the earlier months.

Chillinefs and fhiverings, the ufual precurfors of fever, introduce the other fymptoms of this difeafe. At an early period there is a fenfe of fulnefs and uneafinefs about the throat. Deglutition is generally more difficult and painful than in the Cynanche Maligna, a difeafewhich, in many of its fymptoms, bears a near refemblance to the prefent. A fcarlet eruption is obferved upon the fkin, which in a few days difappears, and where the difeafe is mild the fever ufually fubfides at the fame time.

One of the patients referred to in the lift, was a child about four years old, to whom the difeafe proved fatal. In this cafe, towards the clofe it affumed much of the appearance of the Cynanche Maligna. The fauces were covered with dark coloured floughs; there was a confiderable difcharge through the nofe, of a dark and offenfive matter, and the refpiration became fo laborious that the patient feemed to die of fuffocation.

*History*

